# *HUA ENHANCER1* Mediates Ovule Development

**DOI:** 10.3389/fpls.2020.00397

**Published:** 2020-04-15

**Authors:** Shuai-Jie Wei, Sen Chai, Rui-Min Zhu, Cun-Ying Duan, Yan Zhang, Sha Li

**Affiliations:** State Key Laboratory of Crop Biology, College of Life Sciences, Shandong Agricultural University, Tai’an, China

**Keywords:** integument, fertility, microRNA, HYL1, female gametophytes

## Abstract

Ovules are female reproductive organs of angiosperms, containing sporophytic integuments and gametophytic embryo sacs. After fertilization, embryo sacs develop into embryos and endosperm whereas integuments into seed coat. Ovule development is regulated by transcription factors (TF) whose expression is often controlled by microRNAs. Mutations of Arabidopsis DICER-LIKE 1 (DCL1), a microRNA processing protein, caused defective ovule development and reduced female fertility. However, it was not clear whether other microRNA processing proteins participate in this process and how defective ovule development influenced female fertility. We report that mutations of *HUA ENHANCER1* (*HEN1*) and *HYPONASTIC LEAVES 1* (*HYL1*) interfered with integument growth. The sporophytic defect caused abnormal embryo sac development and inability of mutant ovules to attract pollen tubes, leading to reduced female fertility. We show that the role of *HEN1* in integument growth is cell-autonomous. Although *AUXIN RESPONSE FACTOR 6* (*ARF6*) and *ARF8* were ectopically expressed in mutant ovules, consistent with the reduction of microRNA167 in *hen1*, introducing *arf6*;*arf8* did not suppress ovule defects of *hen1*, suggesting the involvement of more microRNAs in this process. Results presented indicate that the microRNA processing machinery is critical for ovule development and seed production through multiple microRNAs and their targets.

## Introduction

Ovule development is critical for seed yield and plant reproduction. A mature ovule is composed of sporophytic cells, i.e., integuments, and a female gametophyte (FG), called embryo sac (Schneitz et al., 1995, 1997; Christensen et al., 1997; Drews et al., 1998; Drews and Yadegari, 2002; Shi and Yang, 2011). The embryo sac forms embryo and endosperm whereas integuments develop into seed coat after fertilization. During ovule development, megasporogenesis establishes a proximal-distal polarity while megagametogenesis ensures the production of FG (Christensen et al., 1997; Drews et al., 1998; Drews and Yadegari, 2002). A critical event during megagametogenesis in Arabidopsis is the asymmetric growth of outer integuments, which finally envelops the embryo sac and leads to the anatrophy of mature ovules (Schneitz et al., 1995, 1997; Drews et al., 1998; Drews and Yadegari, 2002). Arabidopsis mutants defective in the asymmetric growth of outer integuments often shows defective formation of FG (Bencivenga et al., 2011; Chevalier et al., 2011; Wang et al., 2016), suggesting sporophytic control of gametophytic development.

A number of transcriptional factors (TFs) regulate integument growth (Colombo et al., 2008). Mutations of *INNER NO OUTER* (*INO*), *ABERRANT TESTA SHAPE* (*ATS*), or *AINTEGUMENTA* (*ANT*) compromised the initiation and growth of integuments, resulting in a loss of the outer-inner integument organization (Elliott et al., 1996; Klucher et al., 1996; Villanueva et al., 1999; McAbee et al., 2006). Genes encoding a few homeodomain proteins, such as *BELL1* (*BEL1*) (Reiser et al., 1995), *PHABULOSA* (*PHB*) (Sieber et al., 2004), and *WUSHEL* (*WUS*) (Gross-Hardt et al., 2002; Lieber et al., 2011), play roles in integument growth and ovule development. Except for the positive regulators of integument growth, ectopic expression of *AUXIN RESPONSE FACTOR6* (*ARF6*) and *ARF8* resulted in the arrest of integument growth (Wu et al., 2006), suggesting that there are negative regulators in this process.

Transcription factors are major targets of microRNAs (miRNAs), which are small RNAs of 20- to 24-nucleotide (nt), produced from pre-miRNA-encoding genes, and sequence-specific regulators of gene expression. Because of key roles of miRNAs in plant growth and responses to environment, their expression, processing, and turnover are tightly regulated (Dong et al., 2008; Rogers and Chen, 2013). Mutations at miRNA processing genes often result in reduced fertility (Lu and Fedoroff, 2000; Schauer et al., 2002; Olmedo-Monfil et al., 2010). Arabidopsis DICER-LIKE 1 (DCL1) is a miRNA processing protein (Kurihara and Watanabe, 2004). A mutant of DCL1, *short integuments* (*sin1*/*dcl1-7*), was identified from a chemical mutagenesis (Robinson-Beers et al., 1992). *sin1*/*dcl1-7* is defective in the asymmetric growth of integuments (Robinson-Beers et al., 1992) during ovule developmental stage 3-I when a functional megaspore (FM) is formed (Schneitz et al., 1995, 1997). Efficient and precise processing of pri-miRNAs requires the interaction between DCL1 and HYPONASTIC LEAVES 1 (HYL1) (Kurihara et al., 2006), a dsRNA-binding protein (Vazquez et al., 2004; Dong et al., 2008). HUA ENHANCER1 (HEN1), a multidomain AdoMet-dependent 2′-O-methyltransferase critical for miRNA biogenesis (Chen et al., 2002; Yu et al., 2005, 2010; Baranauske et al., 2015), act in the same pathway as DCL1 and HYL1 (Yang et al., 2010; Baranauske et al., 2015). Whether they participate in ovule development and how their mutations influence female fertility are not clear.

We report here that *hen1-8*, a hypomorphic mutant of *HEN1* (Chen et al., 2002; Yu et al., 2005, 2010), and *hyl1-2*, a null mutant of *HYL1* (Han et al., 2004; Kurihara et al., 2006; Dong et al., 2008), are defective in ovule development. Mutant ovules failed to have asymmetric integument growth, leading to abnormal embryo sac development, compromised pollen tube guidance, and thus reduced female fertility. Downregulating *HEN1* specifically in outer integuments phenocopied *hen1-8*, suggesting a cell-autonomous action. Ectopic expression of *ARF6* and *ARF8* and distorted auxin maximum in *hen1-8* ovules are consistent with the reduction of miRNA167, whose processing relies on HEN1 (Yu et al., 2010; Ren et al., 2012). However, introducing the *arf6*;*arf8* double mutant did not suppress ovule defects of *hen1-8*, suggesting the involvement of more microRNAs in this process.

## Materials and Methods

### Plant Growth and Transformation

Arabidopsis mutants lines, including *hen1-8* (Yu et al., 2010), *hen1-2* (Chen et al., 2002), *hyl1-2* (Vazquez et al., 2004), *arf6-1* (Okushima et al., 2005), *arf8-3* (Nagpal et al., 2005), the transgenic line *Pro_*LAT*__52_*:GUS (Li et al., 2013), DR5:GFP (Ulmasov et al., 1997), PIN1:GFP (Benkova et al., 2003), and *Pro_*ES*__1_*:NLS-YFP (Bencivenga et al., 2012) were described previously. Col-0 ecotype or Landsberg *erecta* (L*er*) was used as the wild type as noted. Arabidopsis plants were grown as described (Zhou et al., 2013). In brief, the seeds were surface-sterilized and then sown on half-strength Murashige and Skoog (MS) basal medium with vitamins (Phytotechlab, SPS0519160A). Plates were placed under 4°C in darkness for 3 days before being moved to a growth chamber with a 16-h-light/8-h-dark cycle at 21°C. One week later, the seedlings were transferred to a 3:1 mix of nutrient soil: vermiculite under same conditions with growth chamber. Plant growth, transformation, and selection were as described (Zhou et al., 2013).

### DNA Manipulation

The artificial miRNA construct targeting *HEN1* (amiR-HEN1) was designed with the primers ZP7781/ZP7782/ZP7783/ZP7784 using WMD3-Designer. The amiR-HEN1 was cloned into pROKII-GFP to generate *Pro_*LAT*__52_*:amiR-HEN1. Later, *Pro_*Lat*__52_* was replaced by *Pro*_*INO*_ to generate *Pro*_*INO*_:amiR-HEN1. The RNAi-HEN1 fragment (2297bp to 2599bp of *HEN1* coding sequence) was amplified with the primer pair ZP6753/ZP6754. The resultant PCR products were sub-cloned into the RNAi vector pTCK303 (Guo et al., 2010) to obtain the *Pro_*UBQ*__10_*:RNAi-HEN1 construct. Later, *Pro_*UBQ*__10_* was replaced by *Pro*_*INO*_ to generate *Pro*_*INO*_:RNAi-HEN1. *Pro_*HEN*__1_* was cloned into pENTR/SD/D-TOPO (Invitrogen) with the primer pair ZP5140/ZP5173, including a 1847 bp sequence upstream of *HEN1* start codon. The entry vector was used in a LR reaction with the destination vector pMD163 (Curtis and Grossniklaus, 2003) to generate *Pro_*HEN*__1_*:GUS. All primers are listed in Supplementary Table S1.

### Genotyping PCRs, RNA Extraction and qPCRs

Genotyping PCRs for *arf6-1* and *arf8-3* were performed using following primer pairs: ZP308/ZP309 and ZP306/ZP307 for the wild copy, ZP1/ZP309 and ZP7546/ZP307 for the mutant copy of *ARF6* and *ARF8*, respectively. Total RNAs were extracted from mature ovules using a Qiagen RNeasy plant mini kit. For qPCRs of *ARF6* and *ARF8* in ovules, oligo(dT)-primed cDNAs were synthesized using a FastQuant RT Kit (TIAN GEN, Cat#KR106-02). Internal controls were as described (Zhou et al., 2013). qPCRs were performed with three biological replicates. Primers used in qPCRs were ZP201/ZP202 for *TUBLIN2*, ZP687/ZP688 for *GAPDH*, ZP7207/ZP7208 for *ARF6*, ZP7209/ZP7210 for *ARF8*, and ZP9325/ZP9326 for *HEN1*. All primers are listed in Supplementary Table S1.

### RNA *in situ* Hybridization

RNA *in situ* hybridization was performed as previously described (Zhou et al., 2013). In brief, the emasculate pistils were fixed in 4% Paraformaldehyde solution (aladdin) at 4°C overnight. Then the fixed tissues were embedded in Paraplast (Sigma-Aldrich) after dehydration and were then sectioned at 8 μm. RNA probes of *ARF6* and *ARF8* were amplified with the primer pairs ZP8093/8094 and ZP8095/8096, respectively. The sense and antisense probes were modified *in vitro* with digoxigenin-UTP by SP6 or T7 RNA polymerases (Roche), respectively. Sections were hybridized with 1.5 ng/μL probes at 42°C overnight in a hybridization solution that contained formamide. Hybridization signals were detected by antidigoxigenin antibody (Anti-Digoxigenin-Ap Fab fragments; Roche). The samples were observed using an Olympus BX53 microscope. All primers are listed in Supplementary Table S1.

### Phenotype Analysis

Pollen tube *in vivo* growth by histochemical GUS staining of *Pro_*LAT*__52_:*GUS-pollinated pistils and aniline blue staining were performed as described (Li et al., 2013). Whole-mount ovule clearing and CLSM of ovules were performed as described (Wang et al., 2016; Liu et al., 2019). Flowers at stage 12 were emasculated and left to grow for 12–16 h before pollination assays.

### Fluorescence Microscopy

Lysotracker red staining was used to show cell silhouettes as described (Wang et al., 2016). CLSM of fluorescence materials was performed with a LSM880 (Zeiss) with the excitation and emission wavelengths set to 488 nm/505–550 nm for YFP and GFP signals and 561 nm/600 nm for RFP signals, respectively.

### Accession Numbers

Arabidopsis Genome Initiative locus identifiers for the genes mentioned in this article are: AT4G20910 for *HEN1*; AT1G09700 for *HYL1*; AT3G22886 for *miRNA167*; AT1G30330 for *ARF6*; AT5G37020 for *ARF8*.

## Results

### *hen1-8* Shows Reduced Fertility Due to Sporophytic Female Defects

**FIGURE 1:**
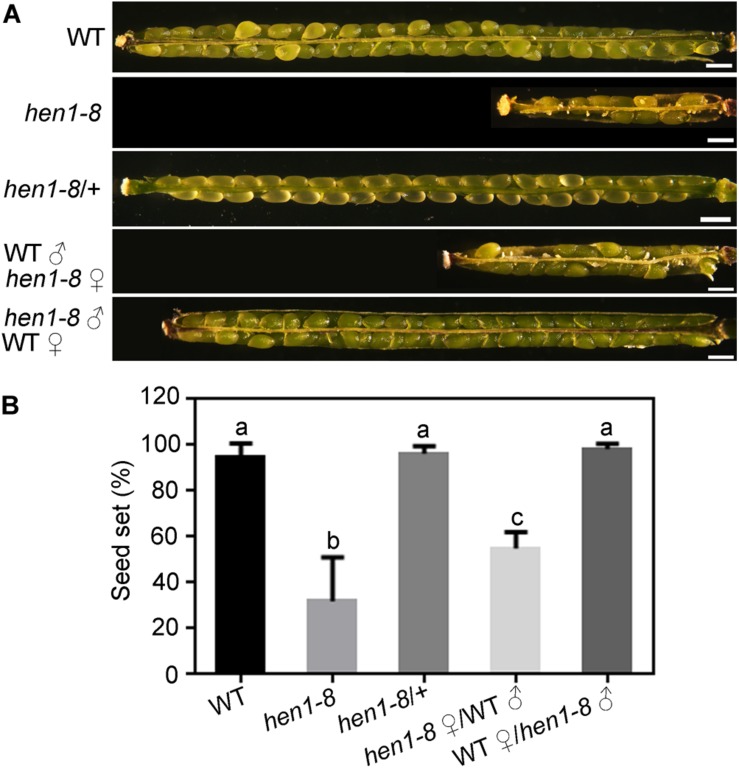
*hen1-8* is significantly reduced in female fertility. **(A)** Representative seed set. For some, two overlapping high-magnification images were taken for one silique and were then overlaid with Photoshop (Adobe Systems) to show the whole silique. **(B)** Quantitative analysis of seed set. Results are means ± standard deviation (SD, *n* = 15). Different numbers indicate significantly different groups (One-Way ANOVA, Tukey’s multiple comparisons test, *P* < 0.05). Bars = 500 μm.

To determine what caused the reduced fertility in *hen1-8* (Chen et al., 2002; Yu et al., 2005, 2010), we performed the following experiments. First, we observed white and wrinkled ovules dispersed among developing seeds in the maturing siliques of *hen1-8* plants, but not in those of wild type or of *hen1-8*/ + (Figures 1A,B), indicating that the reduced fertility is sporophytic. Indeed, segregation ratio by reciprocal crosses indicated that both the male and female gametophytes of *hen1-8* were transmitted normally (Supplementary Table S2). Pollen development of *hen1-8* is also comparable to that of wild type (Supplementary Figure S1). By dissecting siliques from crosses between wild type and *hen1-8*, we observed reduced fertility only when *hen1-8* was used as the female parent (Figures 1A,B), suggesting that the reduced fertility of *hen1-8* was due to sporophytic female defects.

### *hen1-8* Is Defective in Sporophytic Control of Ovule Development

**FIGURE 2:**
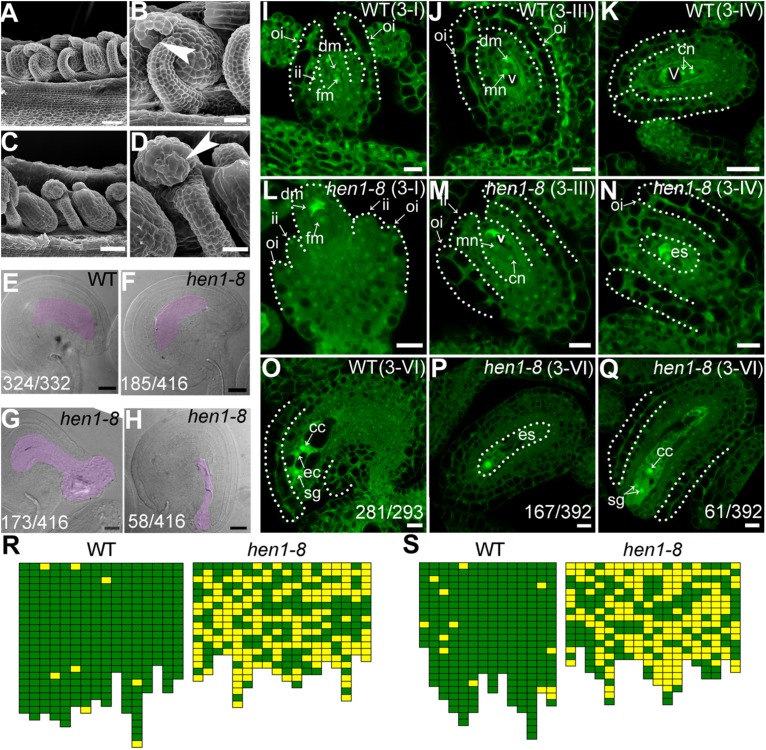
*hen1-8* is defective in ovule development. **(A–D)** Scanning electron micrographs (SEMs) of mature ovules from wild type **(A,B)** or from *hen1-8* **(C,D)**. **(B)** and **(D)** are close-ups of **(A)** and **(C)**, respectively. Arrows in **(B,D)** point at the micropyle. **(E–H)** Whole-mount ovule clearing of wild type **(E)** or *hen1-8* **(F–H)**. The ovule in **(F)** or in **(G–H)** represents normal or abnormal types, respectively. Embryo sacs are highlighted with lilac. **(I–Q)** Confocal laser scanning microscopy (CLSM) of wild-type **(I–K,O)** or *hen1-8* **(L–N,P,Q)** ovules at stage 3-I **(I,L)**, 3-III **(J,M)**, 3-IV **(K,N)**, or 3-VI **(O–Q)**. Ovules were stained with PI and mid-optical sections are shown. For **(I–Q)**, only *hen1-8* ovules with visible nuclei were documented. For **(E–H)** and **(O–Q)**, numbers on the bottom of each image indicate displayed ovules/total ovules examined. Cc, central cell; cn, chalazal nucleus; dm, degenerating megaspore; ec, egg cell; es, embryo sac; fm, functional megaspore; ii, inner integument; oi, outer integument; mn, micropylar nucleus; sg, synergid cell; v, vacuole. **(R–S)** Quantitative analysis of ovule development by ovule clearing **(R)** or by optical sections **(S)**. Each pistil examined was represented by two neighboring columns; the number of cubes in each column indicates the number of countable ovules; normal and abnormal ovules **(R)** or embryo sacs **(S)** are displayed in green and yellow, respectively. Bars = 50 μm for **(A,C)**; 20 μm for **(B,D,E–H)**; 10 μm for **(I–J,L–Q)**; 20 μm for **(K)**.

To determine the reason for sporophytic female defects that caused reduced fertility in *hen1-8*, we examined the morphology of mature ovules by scanning electron micrographs (SEMs) and whole-mount ovule clearing (Wang et al., 2016). At maturation, wild-type ovules showed a typical anatropy with the micropyle proximal to the funiculus (Figures 2A,B). An embryo sac was clearly seen in the mature ovule of wild type (Figure 2E). By contrast, micropyle structure was not discernible in a portion of *hen1-8* ovules (Figures 2C,R). Instead, a bulge, likely a deformed embryo sac, was exposed (Figures 2D,G,H). These results suggested that ovule development is compromised in *hen1-8*.

To determine at which stage the *hen1-8* ovules started to be defective, we performed confocal laser scanning microscopy (CLSM) of developing ovules. At early stages, i.e., before the meiosis of megaspore mother cell (MMC), *hen1-8* and wild type are comparable although *HEN1* is expressed in ovules throughout development (Supplementary Figure S2). However, at stage 3-I when the outer integuments of wild type started rapid and asymmetric growth, extending above the inner integuments (Figure 2I), the growth of *hen1-8* outer integuments was delayed, hardly reaching the length of the inner integuments (Figure 2L). At this stage, functional megaspore (FM) was formed both in wild type and in *hen1-8* (Figures 2I,L). In wild type, from stage 3-III to maturation, the outer integuments continued extended growth, finally enclosing the inner integuments (Figure 2J,K). Every mature ovules of wild type contains an embryo sac with a central cell, an egg cell and two synergid cells (Figure 2O). By contrast, the outer integuments of *hen1-8* failed to enclose the inner structure (Figures 2M,N). At maturation, these ovules contain embryo sacs with abnormal cellular structures such that only one nucleus was visible (Figures 2P,Q,S).

To provide further evidence that *HEN1* is the causative gene for the observed ovule defects in *hen1-8*, we performed additional experiments. Because *hen1-2* is an allelic *HEN1* mutant in *Landsberg errecta* (*Ler*) that contains exactly the same site mutation as in *hen1-8* (Yu et al., 2010), we first examined ovule development of *hen1-2* by CLSM. Indeed, *hen1-2* showed the same ovule defects as those of *hen1-8* (Supplementary Figure S3). Next, we crossed *hen1-8* and *hen1-2* and examined ovules of the F1 progenies. Ovules of the F1 progenies from the cross showed exactly the same defects (Supplementary Figure S4). These results indicated defective outer integument growth affected embryo sac development when *HEN1* is mutated.

### *hen1-8* Ovules Showed Reduced Pollen Tube Attraction

CLSM of *Pro_*ES*__1_*:NLS-YFP;*hen1-8* in which a nucleus-targeted YFP was driven by an embryo sac-specific promoter (Pagnussat et al., 2009), often showed one nucleus, sometimes no nucleus at all, in the embryo sac in contrast to the eight nuclei structure in wild-type ovules (Supplementary Figure S5), suggesting that embryo sac development was compromised due to sporophytic defects in *hen1-8*. This result is also consistent with those obtained by optical section of mature *hen1-8* ovules (Figure 2).

**FIGURE 3:**
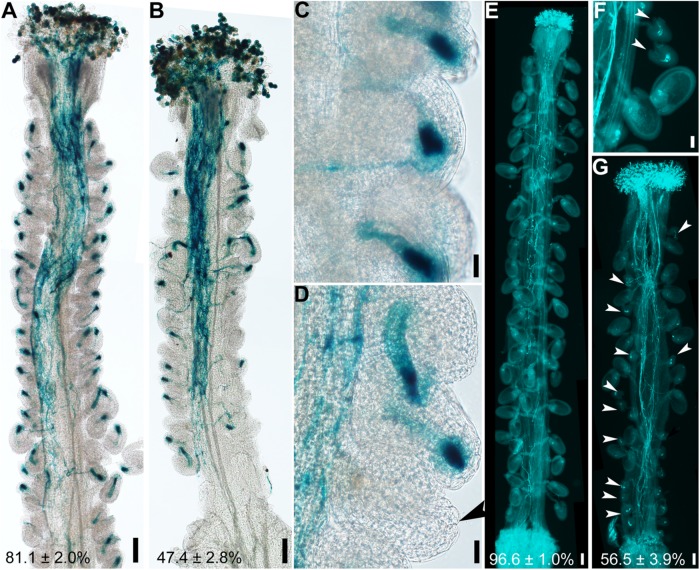
*hen1-8* ovules are compromised in pollen tube guidance. **(A–D)** Histochemical GUS staining of wild-type **(A,C)** or *hen1-8* **(B,D)** pistils at 12 h after pollination (HAP) with *Pro_*LAT*__52_*:GUS pollen. Arrowheads point at an ovule that failed to attract pollen. **(E–G)** Aniline blue staining of wild-type **(E)** or *hen1-8* **(F–G)** pistils at 48 HAP with wild-type pollen. Arrowheads point at ovules that did not develop as a result of fertilization failure. Two to three overlapping high-magnification images were taken for one pistil and overlaid with Photoshop (Adobe) to show the whole pistil **(A,B,E,G)**. Numbers at the bottom of **(A)**, **(B)**, **(C)**, **(D)** are quantification of targeted ovules out of total ovules. Results are means ± SD (*n* = 15). Bars = 100 μm for **(A,B,E,G)**, 20 μm for **(C,D,F)**.

Because the embryo sac within an ovule attracts pollen tubes for fertilization (Higashiyama and Yang, 2017), abnormal embryo sacs due to defective integument growth might be the reason for reduced female fertility in *hen1-8* (Figure 1). To test this hypothesis, wild-type or *hen1-8* pistils were emasculated, and hand-pollinated with *Pro_*LAT*__52_*:GUS pollen and pollen tube attraction at 12 h after pollination (HAP) was examined by histochemical GUS staining. In contrast to wild type in which almost all ovules were targeted by a pollen tube, as indicated by a blue blob inside embryo sacs (Figures 3A,C), over half of *hen1-8* ovules failed to attract a pollen tube (Figures 3B,D). By aniline blue staining of pistils at 48 HAP, we determined that most wild-type ovules were fertilized as indicated by size increase (Figure 3E). By contrast, around half of *hen1-8* ovules were not targeted by pollen tubes and were not fertilized (Figures 3F,G). Therefore, we concluded that defective embryo sac development in *hen1-8* resulted in its reduced female fertility.

### *hyl1-2* Mimicked Ovule Defects of *hen1-8*

**FIGURE 4:**
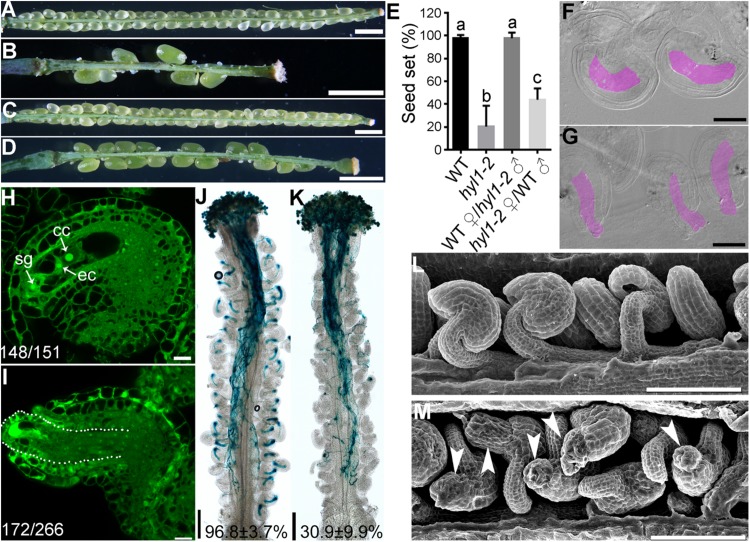
*hyl1-2* is defective in ovule development, similar to *hen1-8*. **(A–D)** Representative silique of wild type **(A)**, *hyl1-2* **(B)**, wild type pollinated with *hyl1-2* pollen **(C)**, or *hyl1-2* pollinated with wild-type pollen **(D)**. **(E)** Quantitative analysis of seed set. Results are means ± SD (*n* = 15). Different numbers indicate significantly different groups (One-Way ANOVA, Tukey’s multiple comparisons test, *P* < 0.05). **(F,G)** Whole-mount ovule clearing of wild type **(F)** or *hyl1-2* **(G)**. Embryo sacs are highlighted with lilac. **(H,I)** CLSM of wild-type **(H)** or *hyl1-2* **(I)** ovules at stage 3-VI. Ovules were stained with PI and mid-optical sections are shown. Numbers on the bottom of each image indicate displayed ovules/total ovules examined. cc, central cell; ec, egg cell; sg, synergid cell. **(J,K)** Histochemical GUS staining of wild-type **(J)** or *hyl1-2* **(K)** pistils at 12 HAP with *Pro_*LAT*__52_*:GUS pollen. Two to three overlapping high-magnification images were taken for one pistil and overlaid with Photoshop (Adobe) to show the whole pistil. Numbers at the bottom are quantification of targeted ovules out of total ovules. Results are means ± SD (*n* = 15). **(L,M)** SEMs of mature ovules from wild type **(L)** or from *hyl1-2* **(M)**. Arrowheads point at ovules with protruding embryo sac. Bars = 1 mm for **(A–D)**; 50 μm for **(F,G)**; 10 μm for **(H–I)**; 200 μm for **(J,K)**; 100 μm for **(L,M)**.

Ovule defects of *hen1-8* were likely due to compromised miRNA processing because HEN1 is critical for the processing of various miRNAs (Yu et al., 2005, 2010; Zhao et al., 2012) and *sin1*/*dcl1-7* showed a similar phenotype (Robinson-Beers et al., 1992). To provide further evidence that the miRNA processing pathway was critical for ovule development, we also examined *hyl1-2* (Vazquez et al., 2004), a null mutant of *HYL1* whose severely reduced fertility was restored to the wild-type level by exogenous *HYL1* (Lian et al., 2013). Female gametophytes of *hyl1-2* transmitted comparably to those of wild type (Xiong et al., 2020), indicating that *HYL1* is not required for the development of female gametophytes. However, the homozygous *hyl1-2* showed a significantly reduced seed set due to sporophytic female defects (Figures 4A–E). By ovule whole-mount analysis (Figures 4F–G), optical sections (Figures 4H–I), and SEM analysis (Figures 4L–M), we demonstrated that *hyl1-2* was defective in ovule development due to the growth arrest of outer integuments. Because of the defects, *hyl1-2* ovules showed a reduced ability to attract pollen tubes compared with those of wild type (Figures 4J–K), leading to significantly reduced female fertility (Figures 4B,D,E).

### HEN1 Functions in a Cell-Autonomous Way

**FIGURE 5:**
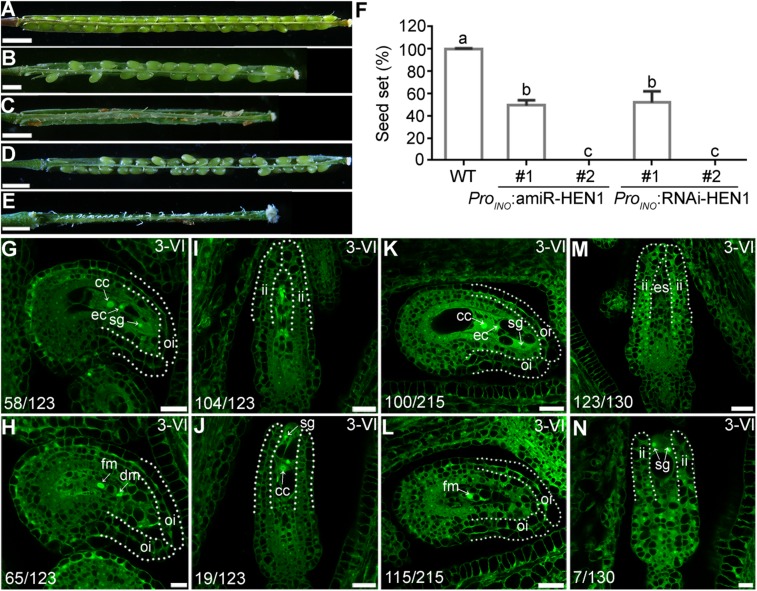
Downregulating *HEN1* in outer integuments mimicked *hen1-8* in ovule development. **(A–E)** Representative silique from wild type **(A)**, two lines of *Pro*_*INO*_:amiR-HEN1 **(B,C)**, and two lines of *Pro*_*INO*_:HEN1-RNAi **(D,E)** transgenic plants. For some pistils, two to three overlapping high-magnification images were taken and overlaid with Photoshop (Adobe). **(F)** Quantification of seed set. Results are means ± SD (*n* = 15). Different numbers indicate significantly different groups (One-Way ANOVA, Tukey’s multiple comparisons test, *P* < 0.05). **(G–N)** CLSM of representative ovules at stage 3-VI from the #1 **(G,H)** or #2 **(I,J)** line of *Pro*_*INO*_:amiR-HEN1, and #1 **(K,L)** or #2 **(M,N)** line of *Pro*_*INO*_:RNAi-HEN1. Numbers on the bottom of each image indicate displayed ovules/total ovules examined. Dotted lines illustrate either outer integuments (oi) or inner integuments (ii). Cc, central cell; ec, egg cell; sg, synergid. Bars = 1 mm for **(A,C,D)**; 500 μm for **(B,E)**; 20 μm for **(G,I,K,M,J,L)**; 10 μm for **(H, N)**.

*hen1-8* shows vegetative growth retardation (Chen et al., 2002), which could have an impact in female fertility. To exclude the possibility that ovule developmental defect of *hen1-8* was resulted from its reduced vegetative growth, we attempted to downregulate the expression of *HEN1* specifically in outer integuments by using the outer integument-specific promoter *Pro*_*INO*_ (Wang et al., 2016). A dozen of transgenic lines containing either *Pro*_*INO*_:amiR-HEN1 (artificial microRNA-*HEN1*) were generated. The transgenic plants were comparable to that of wild type regarding the growth of vegetative tissues (Supplementary Figure S6), consistent with the use of outer-integument-specific promoter. However, seed set of the transgenic plants was compromised (Figures 5B,C,F). We examined two independent transgenic lines representing mildly or severely affected types by CLSM. In the line of *Pro*_*INO*_:amiR-*HEN1* in which seed set was reduced by 50% (Figures 5B,F), half of the mature ovules showed abnormal number of nuclei in their embryo sacs while the integuments were morphologically indistinguishable from those of wild type (Figures 5G,H). In the line where there was hardly any seed set (Figures 5C,F), most mature ovules had no discernible outer integuments or nucleus structure in their embryo sacs (Figures 5I,J).

Because the specificity of *Pro*_*INO*_ used to downregulating *HEN1* (Wang et al., 2016) and the constitutive expression of *HEN1* in ovules (Supplementary Figure S2), we could not verify the downregulation of *HEN1* in the *Pro*_*INO*_:amiR-*HEN1* transgenic plants by quantitative real-time PCRs (qPCRs). Instead, we performed two experiments to support that HEN1 mediates integument growth in a cell-autonomous way. First, we generated *Pro_35__*S*_*:amiR-*HEN1* transgenic plants and examined the transcript abundance of *HEN1* in transgenic seedlings by qPCRs. In randomly selected two *Pro_35__*S*_*:amiR-*HEN1* independent lines, *HEN1* abundance was significantly reduced compared to that in wild type (Supplementary Figure S7), suggesting that the amiR-*HEN1* expression did reduce the mRNA level of *HEN1*. Consistently, the transgenic plants were shorter and smaller than wild-type plants (Supplementary Figure S7). Second, we used a RNA interference (RNAi) approach instead of amiR to downregulate *HEN1* specifically in outer integuments. The *Pro*_*INO*_:*HEN1*-RNAi transgenic plants phenocopied *Pro*_*INO*_:amiR-*HEN1* in reduced seed set (Figures 5D,E,F) and defective ovule development (Figures 5K–N). These results suggested that *HEN1* mediates outer integument growth in a cell-autonomous way.

### Auxin Distribution but Not the Asymmetric PIN1 Localization Was Compromised in *hen1-8* Ovules

**FIGURE 6:**
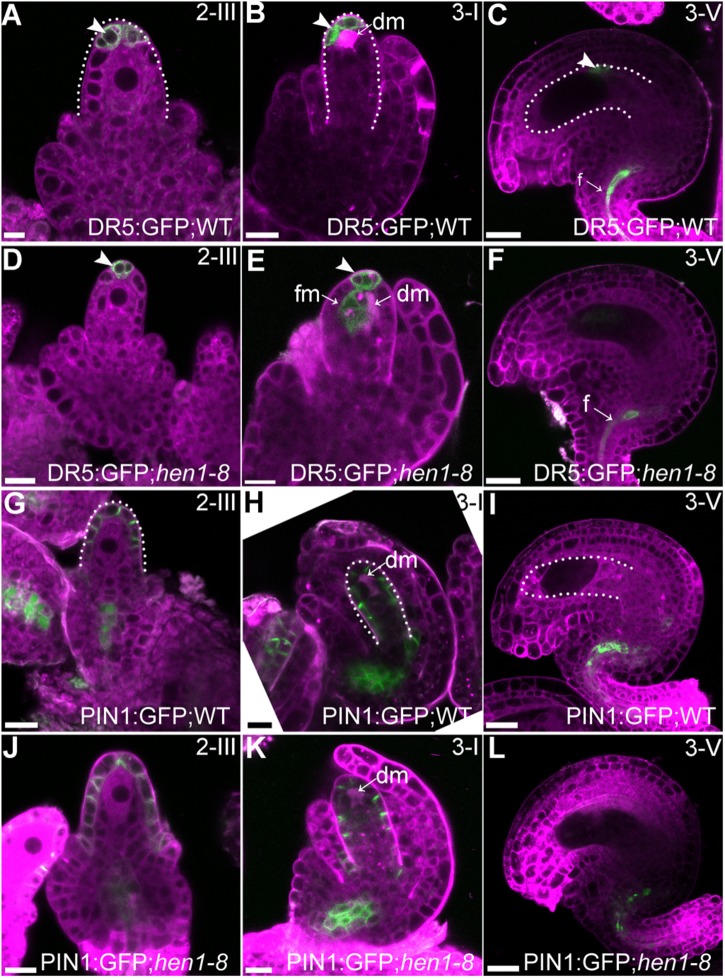
Auxin responses but not the asymmetric PIN1 localization were compromised in *hen1-8* during ovule development. **(A–F)** DR5:GFP in wild-type **(A–C)** or in *hen1-8* **(D–F)** ovules at different stages by CLSM. Arrowheads indicate DR5 signals. **(G–L)** PIN1:GFP in wild-type **(G–I)** or in *hen1-8* **(J–L)** ovules at different stages by CLSM. Ovules were stained with lysotracker red (magenta). dm: degenerating megaspore. Bars = 10 μm.

Auxin is a determinant factor in ovule development (Benkova et al., 2003; Bencivenga et al., 2012; Ceccato et al., 2013). Both auxin receptors and response factors are regulated by miRNAs whose processing depends on the DCL1-HEN1-HYL1 pathway (Navarro et al., 2006; Gutierrez et al., 2009; Ren et al., 2012; Zhao et al., 2012). Therefore, we examined auxin responses by introducing DR5:GFP (Ulmasov et al., 1997) into *hen1-8* and examining GFP distribution. In wild type, GFP signals were detected only in the epidermal cell layer of the nucellus at stage 2-III when both outer and inner integuments were initiated (Figure 6A) and at stage 3-I when outer integuments underwent rapid growth to establish ovule anatrophy (Figure 6B). At maturation, GFP signals were hardly visible in ovules except in the vascular tissues of the funiculus (Figure 6C). The GFP distribution of *hen1-8* was similar to, albeit weaker than, that of wild type at early stages (Figure 6D) and at maturation in morphologically normal *hen1-8* ovules (Figure 6F). However, at stage 3-I, auxin maximum was expanded from the nucellus to the developing female gametophytes of *hen1-8* ovules (Figure 6E), suggesting a spatially disturbed auxin response.

Because PIN1 is the key auxin efflux carrier responsible for auxin distribution during ovule development (Ceccato et al., 2013), we also generated the PIN1:GFP;*hen1-8* plants to examine its distribution. As reported previously (Ceccato et al., 2013; Wang et al., 2016), PIN1 was asymmetrically distributed at the epidermal cells of the nucellus during ovule development (Figures 6G–H) and restricted to the funiculus at maturation (Figure 6I). No difference of PIN1 distribution was observed between wild type and *hen1-8* (Figures 6J–L), indicating that compromised auxin maximum in *hen1-8* ovules was likely resulted from signaling rather than auxin transport.

### Suppressing the Ectopic Expression of *ARF6* and *ARF8* by Introducing *arf6*;*arf8* Did Not Rescue Ovule Defects of *hen1-8*

Mutations of *DCL1* (Robinson-Beers et al., 1992), *HEN1*, and *HYL1* resulted in defective ovule development, suggesting a role of miRNAs in this process. Among miRNAs whose accumulation relies on HEN1 (Ren et al., 2012; Zhao et al., 2012), miRNA167 was demonstrated a positive regulator for ovule development by suppressing the expression of *ARF6* and *ARF8* (Wu et al., 2006; Yao et al., 2019). Indeed, ovule development of the *mir167* mutants was largely recovered by introducing either *arf6* or *arf8* (Yao et al., 2019).

**FIGURE 7:**
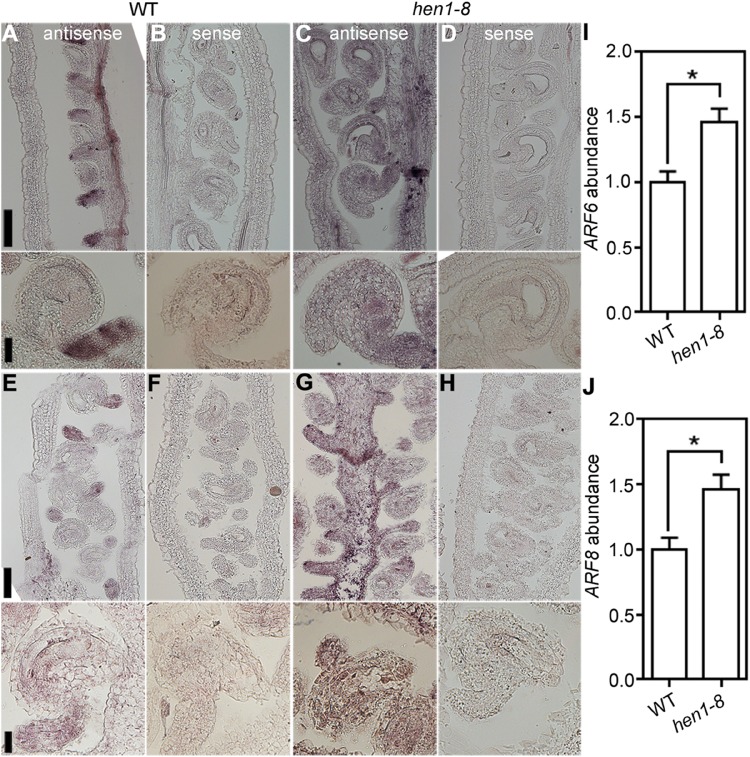
*ARF6* and *ARF8* are ectopically expressed in *hen1-8* ovules. **(A–D)** Stage 3-IV wild-type **(A,B)** or *hen1-8* **(C,D)** ovules labeled by the *ARF6* antisense probe **(A,C)** or sense probe **(B,D)**. **(E–H)** Stage 3-IV wild-type **(E,F)** or *hen1-8* **(G,H)** ovule labeled by the *ARF8* antisense probe **(E,G)** or sense probe **(F,H)**. Funiculus of wild-type ovules whereas integuments of *hen1-8* ovules show strong signals by the antisense probes. **(I,J)** Relative *ARF6* **(I)** or *ARF8* **(J)** abundance in mature ovules of wild type or *hen1-8* by qRT-PCRs. *GAPDH* and *TUBULIN2* were used as internal controls. Results shown are means ± SE (*n* = 3). Asterisks indicate significant difference (*t*-test, *P* < 0.01). Bars = 50 μm for whole views of pistils and 20 μm for close-ups of ovules.

To test the possibility that ectopic expression of *ARF6* and *ARF8* resulted in the arrest of integuments in *hen1-8*, we performed the following experiments. First, we examined the expression of *ARF6* and *ARF8* in *hen1-8* ovules by RNA *in situ* hybridization. As reported previously (Wu et al., 2006), *ARF6* (Figures 7A,B) and *ARF8* (Figures 7E–F) were highly expressed in the funiculus during ovule development in wild type. By contrast, signals of either *ARF6* (Figures 7C,D) or *ARF8* (Figures 7G,H) were detected in whole ovules of *hen1-8*, indicating its ectopic expression. Second, by quantitative RT-PCR (qRT-PCR), we could verify that transcript abundance of both *ARF6* and *ARF8* was significantly increased in *hen1-8* ovules (Figures 7I,J). Third, we introduced the mutants of *ARF6* and *ARF8*, i.e., *arf6-1* and *arf8-3* respectively, into hen1-8 and analyzed the resultant hierarchy mutants. Introducing *arf6-1* or *arf8-3* alone into *hen1-8* did not affect vegetative growth whereas the *arf6-1*;*arf8-3*;*hen1-8* showed a severe growth retardation (Supplementary Figure S8). Close examination of mature ovules from different genotypes showed that either *arf6-1*, or *arf8-3*, or the *arf6-1*;*arf8-3* double mutant could not restore ovule developmental defects of *hen1-8* (Supplementary Figure S9), suggesting that ectopic expression of *ARF6* and *ARF8* was not the reason for the arrest of hen1-8 integuments.

## Discussion

We demonstrate here that mutations of *HEN1* and *HYL1* resulted in the arrest of integument growth during ovule development, similar to those of *DCL1*. Because female gametophyte of *hen1-8* (Supplementary Table S2) and *hyl1-2* (Xiong et al., 2020) transmits comparably that of wild type, the embryo sac defects observed in *hen1-8* and *hyl1-2*, possibly also in *sin1*/*dcl1-7* (Robinson-Beers et al., 1992), were resulted from sporophytic defects through intercellular signaling (Bencivenga et al., 2011), as being reported (Wang et al., 2016; Liu et al., 2019). Embryo sacs send signals to guide directional pollen tube growth (Higashiyama and Yang, 2017). It is therefore understandably that *hen1-8* or *hyl1-2* ovules showed a significantly reduced ability to attract pollen tubes (Figures 3, 4). *hen1-8* is a hypomorphic allele (Yu et al., 2010) whereas *hyl1-2* is a null allele (Han et al., 2004; Kurihara et al., 2006; Dong et al., 2008), which explains the different severity of ovule defects (Figures 1, 4).

A recent study reported that the mutations of *HYL1*, *DCL1*, or *HEN1* caused a reduced number of pollen and megaspore mother cells (Oliver et al., 2017). Our results strongly suggested that fertility reduction in *hen1-8* was due to abnormal ovule development (Figure 3). First, *hen1-8* as the pollen donor to wild type resulted in a full seed set (Figure 1), indicating normal pollen function. Second, pollen development is comparable between *hen1-8* and wild type, despite the relatively small size of *hen1-8* anthers (Supplementary Figure S1). Third, the heterozygous *hen1-8* mutant also produced full seed set (Figure 1), arguing against female gametophytic defects. Indeed, reciprocal crosses between wild type and the heterozygous *hen1-8* indicated that both male and female transmission of *hen1-8* are normal (Supplementary Table S1). The discrepancy between the previous report (Oliver et al., 2017) and ours might be due to the fact that *hen1-8* and *hen1-2* are weaker alleles of *HEN1*.

Despite that the *hen1-8* plants showed sub-optical vegetative growth (Chen et al., 2002; Ren et al., 2012), we believe that *HEN1* works in a cell-autonomous way to regulate the asymmetric growth of outer integuments. Downregulating *HEN1* specifically in outer integuments was sufficient to cause ovule defect similar to, even more severe than, that of *hen1-8* (Figure 5), without affecting vegetative growth (Supplementary Figure S6). Because the cell-specific feature of these transgenic lines, it is difficult, if possible, to examine the reduction of *HEN1* transcript abundance. To make it additionally difficult, in the more severely affected RNAi or amiR lines, the growth of outer integuments was arrested very early on. But the two different constructs used to downregulating *HEN1* in outer integuments gave the same results, strongly supporting a cell autonomous role of *HEN1* in outer integuments.

The accumulation of miRNA167 was significantly reduced in *hen1* mutants (Yu et al., 2010; Ren et al., 2012). Consistently, *ARF6* and *ARF8*, major targets of miRNA167 (Nagpal et al., 2005; Wu et al., 2006; Yao et al., 2019; Zheng et al., 2019), were ectopically expressed in *hen1-8* (Figure 7). However, introducing *arf6* or *arf8* did not suppress developmental defects of *hen1-8* ovules (Supplementary Figure S9). The inability is unlikely to have caused by substantially compromised growth of the *arf6-1*;*arf8-3*;*hen1-8* triple mutant since introducing either *arf6-1* or *arf8-3* didn’t aggravate the growth of *hen1-8* but yet was not able to rescue its defects (Supplementary Figure S8). A more likely possibility is that more miRNAs downstream of HEN1 play roles in this process. Auxin maximum was altered in developing ovules of *hen1-8* such that DR5 signals were expanded to the developing female gametophytes in *hen1-8* rather than restricted to the nucellus as in wild type (Figure 6). Because the sporophytic integuments affect FG development (Bencivenga et al., 2011; Wang et al., 2016; Liu et al., 2019), these results indicated that auxin signaling in integuments was compromised in *hen1-8*. Indeed, genes encoding auxin receptors are also targets of miRNAs (Navarro et al., 2006; Gutierrez et al., 2009). A genome-wide small RNA sequencing will be useful to identify miRNAs that are expressed in integuments and whose reduced levels result in the arrest of integument growth in mutants of the DCL1-HEN1-HYL1 pathway.

## Data Availability Statement

All datasets generated for this study are included in the article/Supplementary Material.

## Author Contributions

S-JW and SC performed all the experiments with the assistance of R-MZ and C-YD. SL and YZ conceived and supervised the project and secured the funding. S-JW, SL, and YZ analyzed the data. YZ wrote the article with input from all authors.

## Conflict of Interest

The authors declare that the research was conducted in the absence of any commercial or financial relationships that could be construed as a potential conflict of interest.
