# Supplementary material for: HUA ENHANCER1 Mediates Ovule Development
Source: Front Plant Sci. 2020 Apr 15;11:397. doi: 10.3389/fpls.2020.00397 (PMC7174553; doi:10.3389/fpls.2020.00397)
Supplement: Supplementary file 1 [file Data_Sheet_1.PDF]

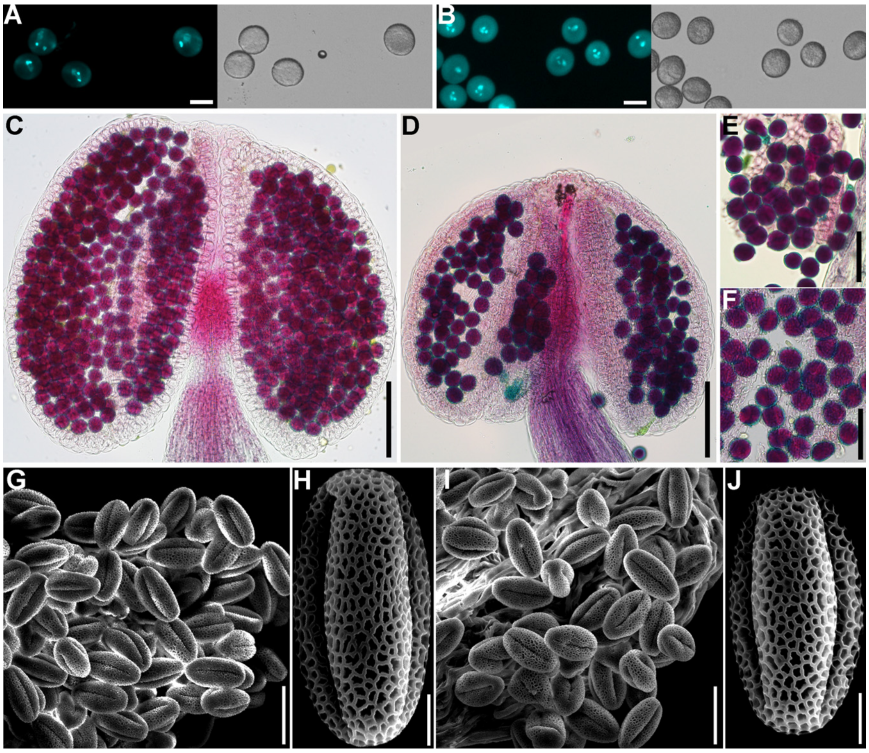

Figure S1. Pollen development is normal in *hen1-8*.

(A-B) DAPI staining of mature pollen grains from wild type (A) or *hen1-8* (B). DAPI channel image is shown at the side of its corresponding transmission image. (C-F) Alexander staining of a dehiscing anther (C, D) or mature pollen grains (E, F) from wild type (C, E) or *hen1-8* (D, F). (G-J) Scanning electron micrographs (SEMs) of a dehiscing anther (G, I) or a mature pollen grain (H, J) from wild type (G, H) or *hen1-8* (I, J). For (C, D, G, H), over 20 anthers were examined with similar results. For (A, B, E, F), over 100 pollen grains were examined with similar results. For (H, J), over 10 pollen grains were examined with similar results. Bars = 20 µm for (A-B); 100 µm for (C-D); 50 µm for (E-F); 25 µm for (G, I); 5 µm for (H, J).

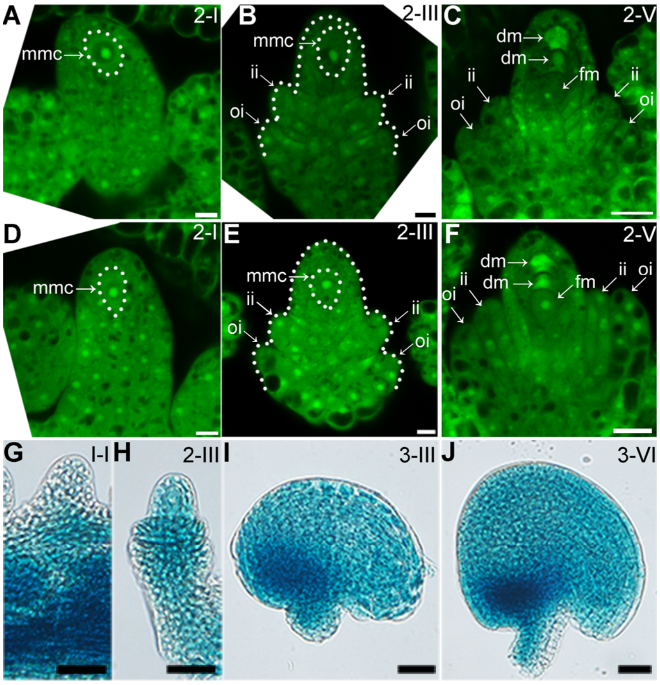

Figure S2. Integument growth of *hen1-8* was comparable to that of wild type at early stages of ovule development.

(A-F) Confocal laser scanning microscopy (CLSM) of wild-type (A-C) or *hen1-8* (D-F) ovules at stage 2-I (A, D), 2-III (B, E), or 2-V (C, F). Ovules were stained with PI and mid-optical sections are shown. dm, degenerating microspores; fm, functional microspore; ii, inner integument; mmc, megaspore mother cell; oi, outer integument. Over 100 ovules from 6 mature pistils were examined with similar results. (G-J) Representative histochemical GUS staining of Pro_{HEN1}:GUS transgenic ovules at different stages. Ten independent transgenic plants were examined with similar results. Bars = 5 µm for (A, B, D, E); 10 µm for (C, F); 20 µm for (G-J) .

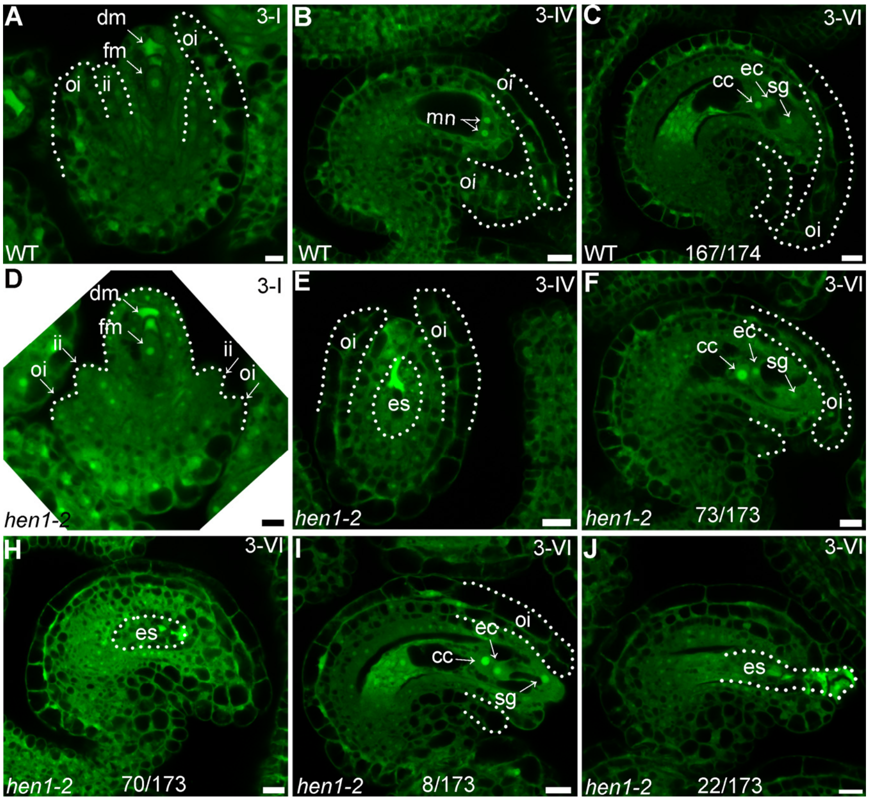

Figure S3. Ovules of *hen1-2* showed similar defects as those of *hen1-8*.

(A-C) Representative CLSM of L*er* ovules (WT) at stage 3-I (A), 3-IV (B), or 3-VI (C). (D-J) Representative CLSM of *hen1-2* ovules at stage 3-I (D), 3-IV (E), or 3-VI (C-J). All four types of hen1-2 ovules, ranging from wild-type-like (F), without clear embryo sac structure (H), with protruding yet well-patterned embryo sacs (I), or with protruding and deformed embryo sacs (J). Number at the bottom indicates displayed ovules/total examined ovules. Cc, central cell; dm, degenerating megaspore; ec, egg cell; es, embryo sac; fm, functional megaspore; ii, inner integument; mn, micropylar nucleus; oi, outer integument; sg, synergid cell. Bars = 5 µm for (A, D); 10 µm for (B, C, E-J).

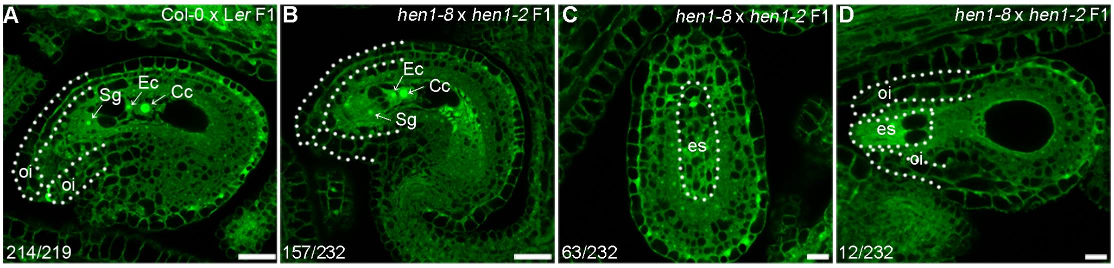

Figure S4. F1 progenies from *hen1-8* and *hen1-2* crosses showed the same ovule defects as their parents.

(A) A representative ovule of F1 progenies from Col-0 and L*er* crosses at maturation. (B-D) Representative ovules of F1 progenies from *hen1-8* and *hen1-2* crosses at maturation. Number at the bottom indicates displayed ovules/total examined ovules. Cc, central cell; Ec, egg cell; es, embryo sac; ii, inner integument; oi, outer integument; Sg, synergid cell. Bars = 20 µm for (A, B); 10 µm for (C, D).

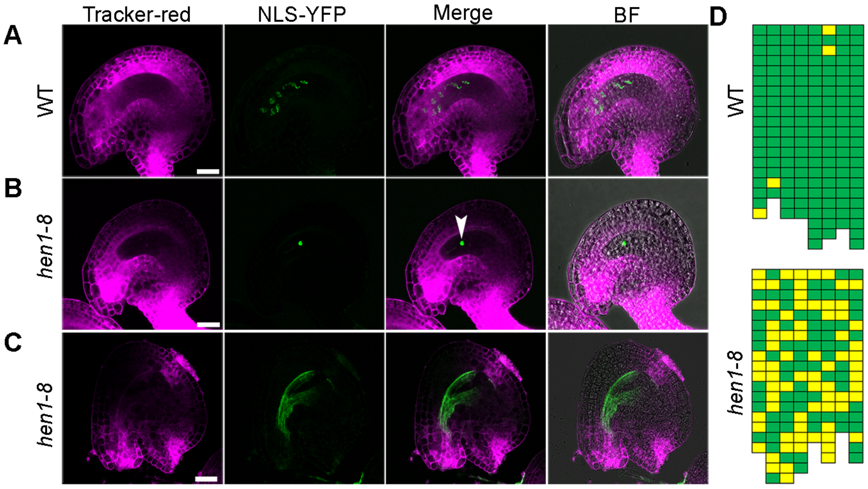

Figure S5. Embryo sac development of *hen1-8* was impaired.
(A-C) Representative CLSM of mature ovules from Pro_{ES1}:NLS-YFP (WT, A) or from Pro_{ES1}:NLS-YFP;*hen1-8* (B-C) plants. Ovules shown in (B) and (C) represent two types of abnormal NLS-YFP patterns. Ovules were stained with lysotracker red (magenta). (D) Quantitative analysis of NLS-YFP patterning in ovules. Each pistil examined was represented by two neighboring columns; the number of cubes in each column indicates the number of countable ovules; normal and abnormal ovules are displayed in green and yellow, respectively. Bars = 20 µm.

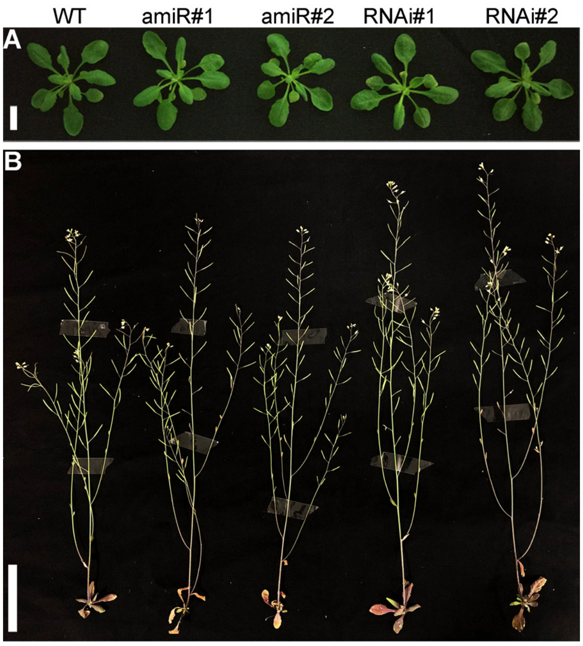

Figure S6. The vegetative growth of *Pro$_{INO}$*:amiR-HEN1 or *Pro$_{INO}$*:HEN1-RNAi transgenic plants is comparable to that of wild type.

(A-B) Representative wild type, two lines of *Pro$_{INO}$*:amiR-HEN1, and two lines of *Pro$_{INO}$*:HEN1-RNAi transgenic plants at 3 WAG (A) or 6 WAG (B). Bars = 1 cm for (A); 5 cm for (B).

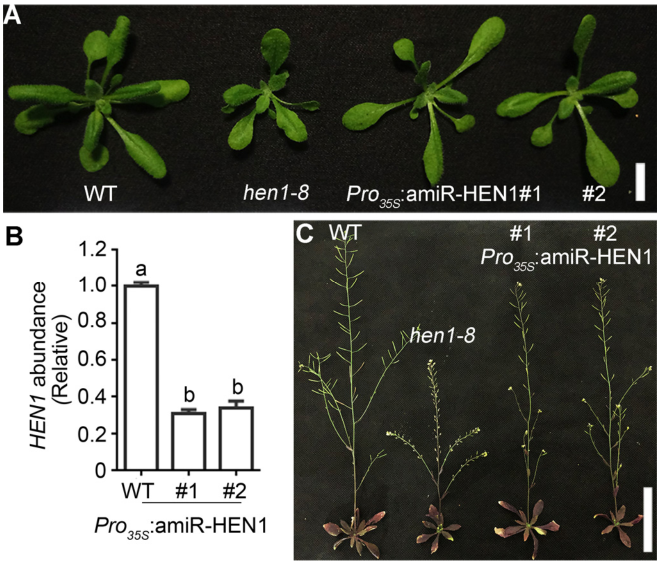

Figure S7. The amiR-*HEN1* downregulates *HEN1* abundance.
(A) Representative wild-type, *hen1-8*, and two lines of *Pro35S:amiR-HEN1* at 3 WAG. (B) Relative transcript abundance of *HEN1* in seedlings of designated genotypes. Results are means ± standard errors (SE, n=3). Different letters indicate significantly different groups (One-Way ANOVA, Tukey's multiple comparison test, $P<0.05$). (C) Representative wild-type, *hen1-8*, and two lines of *Pro35S:amiR-HEN1* at 7 WAG. Bars = 1 cm for (A); 5 cm for (C).

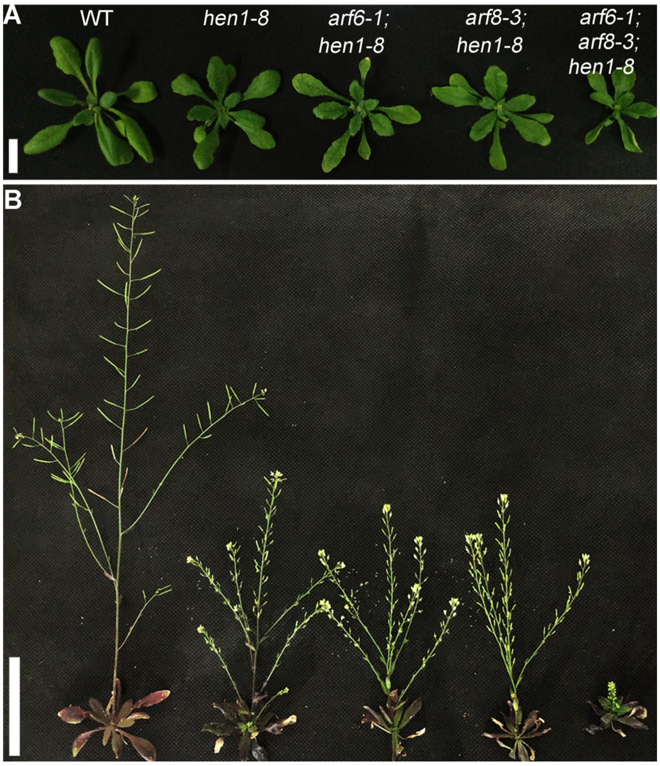

Figure S8. Vegetative growth of *hen1-8* was further compromised by functional loss of *ARF6* and *ARF8*.

(A-B) Representative wild type, *hen1-8*, *arf6-1;hen1-8*, *arf8-3;hen1-8*, and *arf6-1;arf8-3;hen1-8* plants at 4 WAG (A) or 7 WAG (B). Bars = 1 cm for (A); 5 cm for (B).

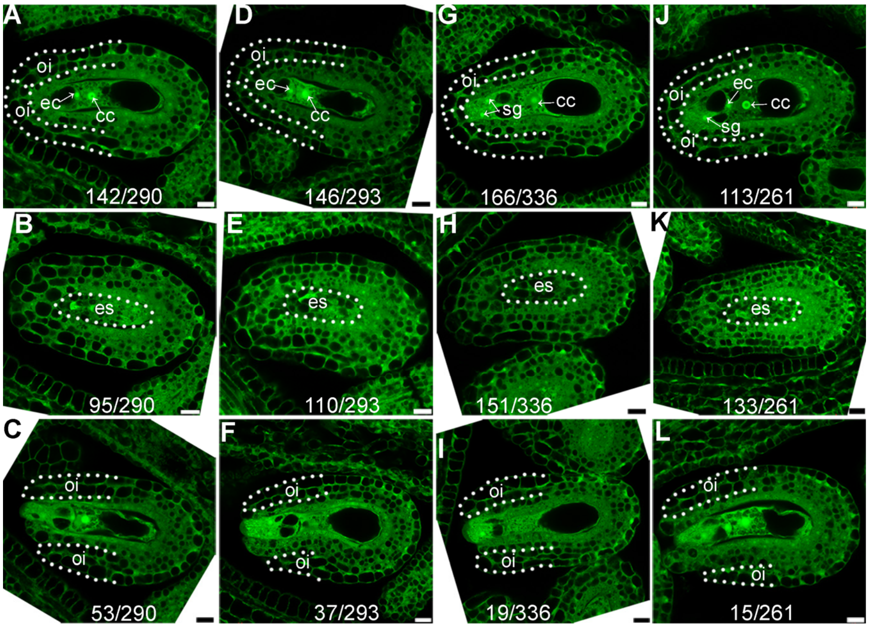

Figure S9. Functional loss of *ARF6* and *ARF8* did not restore ovule development in *hen1-8*.

(A-L) CLSM of *hen1-8* (A-C), *arf6-1*;*hen1-8* (D-F), *arf8-3*;*hen1-8* (G-I), or *arf6-1*;*arf8-3*;*hen1-8* (J-L) ovules at maturation. Ovules were stained with PI and mid-optical sections are shown. Number at the bottom is displayed ovules/total examined ovules of a given genotype. Cc, central cell; cn, chalazal nucleus; ec, egg cell; es, embryo sac; ii, inner integument; oi, outer integument; mn, micropylar nucleus; sg, synergid cell. Bars = 10 μm.

Table S1. Oligos used in this study.

Application		No.	5'-3' sequence
qRT-PCR	*TUBLIN2*	ZP201	ATCCGTGAAGAGTACCCAGAT
		ZP202	AAGAACCATGCACTCATCAGC
	GAPDH	ZP687	TGAAATCAAAAAGCTATCAAGG
		ZP688	CATCATCCTCGGTGTATCCAA
	ARF6	ZP7207	TTCTTAATTCTGAGCTCTGGCA
		ZP7208	ACTTCATCAGTCTCCACATCAG
	ARF8	ZP7209	CATTAAAGCCGTTTTTCACACG
		ZP7210	TTATAGTACCCATGTACCTGCG
	HEN1	ZP9325	GAAATCGTGGAAGCACTAGCTAC
		ZP9326	CAATACTATCCAAGTAATGCCGG
Clone	*amiR-HEN1*	ZP7781	GATTATATATACAGCCGCGACTTTCTCTCTTTTGTATTCC
		ZP7782	GAAAGTCGCGGCTGTATATATAATCAAAGAGAATCAATGA
		ZP7783	GAAAATCGCGGCTGTTTATATATTCACAGGTCGTGATATG
		ZP7784	GAATATATAAACAGCCGCGATTTTCTACATATATATTCCT
	RNAi-HEN1	ZP6753	ATATGGATCCGAGCTCCTTGCAACGTCAAATCTGCTAC
		ZP6754	ATATGGTACCACTAGTCTCTTGTCCACTCGAATTTGTG
	Pro_{HEN1}	ZP5140	CACCTCATGGATTCGTGGTATAGCGTTACTT
		ZP5173	CACAAACACAATGTAGCTTCTTTAACAC
RNA *in situ*	*ARF6*	ZP8093	GAATTGATTTAGGTGACACTATAGAGGCATTGATCCTGCAAAAG'
		ZP8094	GAATTGTAATACGACTCACTATAGGGAAGGTTTGACATTCCGTTCG
	ARF8	ZP8095	GAATTGATTTAGGTGACACTATAGGAAGGGGTGATTTGGGAAGT
		ZP8096	GAATTGTAATACGACTCACTATAGGGGGTTGGACGAGTTAATCTGTCC
Genotyping PCRs		ZP306	CTAGATTCTGTTCGTTGG
		ZP307	TGAGGCTCTTGCAGCTGT
		ZP308	GACGAATCTACTGCAGGAG
		ZP309	TTGGGCTGAGAAGCAGAA
		ZP1	ATTTTGCCGATTTCGGAAC
		ZP7546	GGCAATCAGCTGTTGCCCGTCTCACTGGTG

Table S2. Male and female transmissions were not impaired in *hen1-8*.

Parents	F1 progenies			Statistics	
Male x Female	Genotype	Expected Ratio	Observed		
hen1-8+/- x wild type	*HEN1*+/+ : +/-	1:1	76:71	x^2=0.0272	x^2<$x^2_{0.05,1}$
wild type x *hen1-8*+/-	*HEN1*+/+ : +/-	1:1	73:79	x^2=0.0329	x^2<$x^2_{0.05,1}$
hen1-8+/- x *hen1-8*+/-	*HEN1*+/+ : +/- : -/-	1:2:1	26:64:27	x^2=0.7948	x^2<$x^2_{0.05,2}$
